# Changes in quality of life among Norwegian school children: a six-month follow-up study

**DOI:** 10.1186/1477-7525-7-7

**Published:** 2009-02-04

**Authors:** Thomas Jozefiak, Bo Larsson, Lars Wichstrøm

**Affiliations:** 1 The Norwegian University of Technology and Science (NTNU), Regional Centre of Child and Adolescent Mental Health, MTFS N-7489, Trondheim, Norway; 2 Department of Child and Adolescent Psychiatry, St Olavs Hospital, N-7433 Trondheim, Norway; 3 The Norwegian University of Technology and Science (NTNU), Department of Psychology, N-7491 Trondheim, Norway

## Abstract

**Background:**

A considerable gap exists in regard to longitudinal research on quality of life (QoL) in community populations of children and adolescents. Changes and stability of QoL have been poorly examined, despite the fact that children and adolescents undergo profound developmental changes. The aims of the study were to investigate short-term changes in student QoL with regard to sex and age in a school-based sample.

**Methods:**

A representative Norwegian sample of 1,821 school children, aged 8–16 years and their parents were tested at baseline and 6 months later, using the Inventory of Life Quality for Children and Adolescents (ILC) and the Kinder Lebensqualität Fragebogen (KINDL). Student response rate at baseline was 71.2% and attrition over the follow-up period was 4.6%, and 1,336 parents (70%) completed the follow-up. Change scores between baseline and follow-up evaluations were analysed by means of ANCOVA in regard to sex and age effects.

**Results:**

Students in the 8^th ^grade reported a decrease in QoL over the six-month follow-up period as compared to those in the 6^th ^grade with regard to Family and School domains and total QoL on the KINDL. For emotional well-being a significant linear decrease in QoL across grades 6^th ^to 10^th ^was observed. However, student ratings on the Friends and Self-esteem domains did not change significantly by age. Girls reported a higher decrease in their QoL across all grades over the follow-up period than did boys in respect of Self-esteem on the KINDL, and an age-related decrease in total QoL between 6^th ^and 8^th ^grade on the ILC. Parent reports of changes in child QoL were nonsignificant on most of the domains.

**Conclusion:**

The observed age and sex-related changes in school children's QoL across the six-month follow-up period should be considered in epidemiological as well as clinical research.

## Background

In spite of no gold standard for the definition of QoL, there is a broad consensus to regard the concept of QoL as multidimensional, covering physical, psychological and social dimensions [1]. Thus, for the purpose of the present study, we have defined "QoL" as "the subjective reported well-being in regard to the child's physical and mental health, self-esteem and perception of own activities (playing/having hobbies), perceived relationship to friends and family as well as to school."

Given the profound developmental changes that occur over relatively short time frames during childhood and adolescence, it is of particular concern that QoL changes in community populations of children and adolescents have been poorly examined. For example, in respect to family-related QoL, the child's relationship to the parents during puberty merits further investigation. So far, dramatic shifts in conflict behaviour as a function of age or maturation in childhood and adolescence have not been found [2]. However, Larson et al. [3] reported that the amount of time that 5^th^–12^th ^grade students spend with their families decreased considerably during this age period, indicating disengagement from parents. According to a transformation model [4] adolescents' affect with family decreases in early adolescence and then increases in late adolescence [3,5]. Thus, an important developmental task for adolescents is to achieve psychological independence from parents, while maintaining connectedness with them [3], possibly having a negative impact on family-related QoL.

The subjective well-being related to friends represents another social life domain often included in QoL assessment of children and adolescents (for an overview of instruments, see Spieth [6] and Eiser [7]). It has been shown that parallel to observed changes in the relationship between the adolescent and his/her family, time spent by the adolescent with friends outside the family increases with increasing age [4,8]. These extrafamilial relationships during adolescence often serve the same functions as familial relationships do during childhood. Intimacy, mutuality and self-disclosure between friends peak during adolescence, when developing relations to significant friends is greater than in other life period [4]. Having friends has been associated with a sense of well-being [9], and for 4^th ^and 8^th ^graders, friendship [10] has been found to be quite stable during a six-month period.

The life domain School represents the third social context of importance in the assessment of QoL in children and adolescents. However, the impact of changes occurring in community populations in the school QoL area is still poorly investigated. Transitions during early adolescence from primary to junior high school may also have a negative influence on the child [11]. School bonding refers to "connections" that young people have with their schools and various aspects of their academic lives. It has been positively linked to student adjustment and perceived school climate, but inversely correlated with levels of problem behaviour [12,13]. School bonding has also been shown to be higher among 6^th ^graders than 7^th ^or 8^th ^graders [13].

The domain Emotional well-being, reflecting normal psychological development in children and adolescents in different social contexts, is often included in QoL assessment of children and adolescents [6], as well as the Self-esteem domain [14-16]. Although an extensive meta-analysis concluded that self-esteem showed substantial continuity and stability over time [17], self-esteem in some children may depend on fluctuating social approval from significant others [18].

Developmental transitions may follow different courses for girls and boys, also in different cultural contexts. For example, only Caucasian girls reported a decline in self-esteem from age 11 to 16 years as compared to black girls [19]. Generally, in cross-sectional studies of QoL in general populations, adolescent girls have reported significantly lower quality of life than younger children and boys [20,21]. To date limited information exists on gender differences and should be further investigated.

While most previous longitudinal research on QoL in children has focused on various somatic diseases such as cancer [22], cerebral palsy [23], epilepsy [24], and brain injury [25], it is important also to evaluate changes of QoL among children and adolescents in the general population, because changes in QoL in clinical populations cannot be adequately understood without such reference data. Such information will serve as reference in research evaluations of drug and psychological interventions [26] for children typically being conducted within a relative short time frame. In a longitudinal study, Shek and colleagues [27,28] examined family life quality in Chinese adolescents, and a school-based study [29] in Australia followed 363 students, primarily girls, aged 10 to 18 years, over a six-month period in order to examine changes in their QoL. The results showed that most of the students reported good to excellent QoL both at baseline and at the follow-up [29]. However, no specific information was provided on QoL changes by group or gender related to developmental issues for adolescents. Overall, the existing knowledge on the extent and type of short-term QoL changes in community populations, and how children's normal development influences their experience of QoL is very limited.

Given the substantial discrepancy between child and parent reports of child QoL in cross-sectional studies [30-36], it has been recommended to include both self and parent by proxy reports in QoL studies of children and adolescents [30,37]. In a recent cross-sectional study [37] we investigated discrepancies between informants, and found that parents in the general population evaluated their children's QoL as higher than did the children themselves.

The aims of the present study were to investigate six-month changes in self- and parent reports of child QoL, related to sex and age, in a representative school-based sample of Norwegian students, aged 8–16 years. It was hypothesized that over the six month follow-up,

(1) increasing age will have a decreasing effect on family-related QoL, school-related QoL and emotional well-being; while the students' perceived relationship to friends and self-esteem will be stable across age-groups.

(2) girls will report lower total QoL levels than boys.

(3) parent by proxy ratings will show fewer significant age and sex-related changes in child QoL than student reports on different life domains.

## Method

### Population, sample selection and subjects

#### The baseline sample

The students in the county were stratified according to geography and grade, and 4^th^, 6^th^, 8^th ^and 10^th ^grades were included. In the county of Sør-Trøndelag, half of the population lives in typical urban (the city of Trondheim), and the other half in rural areas. Almost all of students attend public primary school, consisting of elementary (1^th ^to 7^th ^grade) and junior high school (8^th ^to 10^th ^grade). Further, in Norwegian elementary school, students do not receive marks. When the data were collected from September 2004 until November 2005, due to a school reform, traditional classes both in elementary and junior high school were dispersed and reorganized in grade cohorts, i.e. all students attending a specific grade received lessons sometimes together or separately in different minor groups.

**Figure 1:**
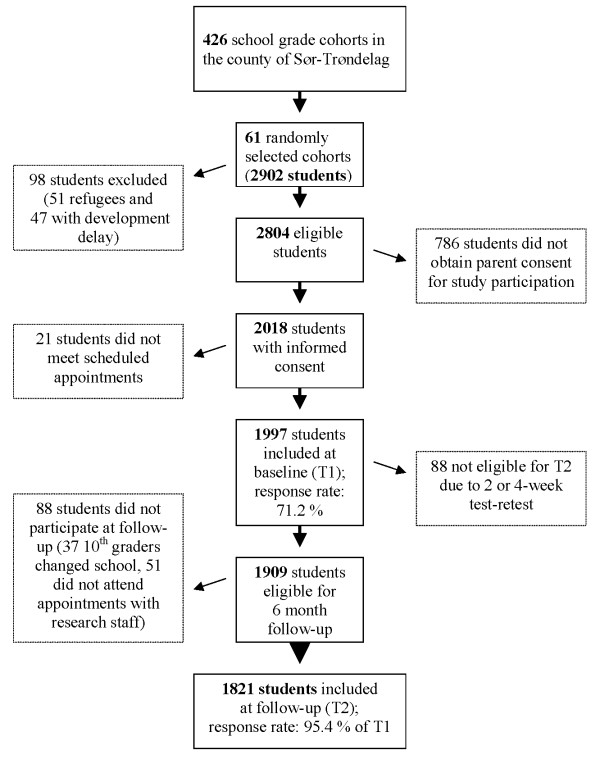
**Flowchart of sample selection**.

The national Norwegian database for primary education (GSI) was used to enumerate all pupils attending any of the targeted grades in all schools and relevant region. Thus, 426 school grade cohorts were identified. Using a cluster sampling technique, 61 were randomly selected for the study (see subject flow in figure 1). Thus, 1,997 students (990 girls and 1,007 boys) aged 8–16 years were finally included in the study, yielding a response rate of 71.2% (of 2804). Table 1 shows the number and age range of included students per grade. For 1,777 (89%) of the 1,997 students, there was at least one caregiver who filled out the Inventory of Life Quality for Children (ILC) [38], and for 1,743 (87%) students at least one caregiver filled out the Kinder Lebensqualität Fragebogen (KINDL) [14,15]. Exclusion criteria for the study were one or more of the following: insufficient competence in the Norwegian language or having a developmental level corresponding to more than two years below the relevant grade. To decide if a student fulfilled the exclusion criteria, the local coordinator (a teacher at each school), discussed possible students being excluded from the study with the principal investigator (the first author).

**Table 1:** Number of subjects by grade and age at baseline and 6-month follow-up

		Baseline	Six-month follow-up
			
Grade	Age (years)	n	n
4^th^	8–10	505	490
6^th^	10–12	462	447
8^th^	12–14	492	383
10^th^	14–16	538	501
Total	8–16	1997	1821

The urban-to-rural residency ratio of the included children in the main study sample was 1:1, compared to 1.2 : 1 in the county. Further, students from 24 of the 25 municipalities in the county were included. The male-to-female ratio was almost identical in the study sample (1.02:1) compared to the county (1.03:1). The mean age of included students was 12.1 (SD = 2.3), and the number of included students per grade ranged from 462 to 538 (see Table 1). Thus, the baseline sample was approximately representative in regard to geography, but also for age, and grade.

The aims of the baseline study [37] were to assess psychometric properties of two translated QoL instruments, the KINDL and ILC, and to investigate factors influencing the degree of discrepancy in regard to child and parent by proxy ratings of child QoL.

#### The six-month follow-up sample

##### Students

Of students eligible for the 6-month follow-up, 1821 (95.4% of the baseline sample) completed the assessment (see Figure 1). This sample was still representative for the population with regard to urban-to-rural resident ratio (1 : 1.1) and sex ratio (1 : 1.01). The number of 8^th ^grade students were reduced (see Table 1) due to attrition and 88 students who were not eligible due to 2 or 4-week test-retest evaluation (see Figure 1). Student mean age was almost identical in the follow-up (Mean 12.0, SD = 2.3) to baseline assessment (Mean 12.1, SD = 2.3). There was no significant difference in total QoL baseline scores on the KINDL between participants and non-participants at the six-month follow-up (Mean = 70.5, SD = 12.5; Mean = 69.1, SD = 13.0, respectively). The mean interval between baseline and follow-up was 180 days (SD = 9.1), and time intervals (< 0.5 SD, ± 0.5 SD and > 0.5 SD) were unrelated to changes in KINDL QoL scores.

##### Parents

At the follow-up, 1,336 students (70% of 1,909 eligible students) had at least one parent who completed the measure. Results of independent t-test showed that parents who participated in both the baseline and follow-up evaluations reported significantly higher KINDL total QoL scale scores at baseline (Mean = 76.4, SD = 9.7) than nonparticipants at the follow-up (Mean 74.8, SD = 9.7), t(1681) = 2.6, p < 0.01.

Given low reliability on some of the KINDL subscales for the youngest children [37], we included 4^th ^graders only on the Self-esteem, Family and Total QoL scales. Due to low test-retest reliability the KINDL Physical well-being scale was not included in the analysis, but was used in calculating KINDL QoL total score for all grades.

### Assessment procedures

One teacher at each school was appointed as a project coordinator and given information about the research project and procedures for collecting the data. The coordinator informed the students about the project and also sent a standard information letter to their parents. The principal investigator (the first author) or a research assistant was present at each school when the students filled out the questionnaires. They stressed informant confidentiality, responded to questions, and read questions aloud for students with reading problems and all pupils in the 4^th ^grade. Completed questionnaires marked with an ID number were collected in sealed envelopes by the researchers. A total of 105 students being absent on the day of data collection at follow-up completed the questionnaires individually during the following week under the supervision of the local coordinator.

### Measures

#### The Inventory of Life Quality in Children and Adolescents (ILC)

The ILC, consists of 15 items [38], and was developed as a short and practical assessment tool for use in child mental health settings. A Norwegian translation of the generic 7-item ILC for children, adolescents and their parents was used to assess QoL over the past week [37]. The ILC includes one global QoL item, and six items addressing school performance, family functioning, social integration, interests and hobbies, physical health, and the child's mental health areas. Each item is rated on a 1 – 5 scale (1 = very good, 5 = very bad). The ILC LQ0-100 score was obtained by summing the 7 items, and transformed into a 0–100 scale in accordance with the originator [38]. Thus, 0 indicates very low and 100 very high QoL.

In school populations, the ILC has shown acceptable internal consistency, with alpha of .63 (alpha = .76 for the parent version). Test-retest reliability was r = .72 for the ILC LQ0-100 score (r = .80 for the parent version) [38]. In a study of German child psychiatric outpatients (N = 728) effect sizes were reported to be d = .30 to .54 for single items in respect of significant QoL changes at a one-year follow-up [39]. The ILC has also shown a moderate convergent validity with the KINDL (r = .65) [14,40]. In the Norwegian translation, student ratings on the ILC LQ0-100 and the KINDL total QoL scale correlated moderately with each other (r = .69). The Norwegian version of the 7-item ILC has shown satisfactory internal consistency for the 7 items (alpha from 0.64 to 0.81 for the 4^th ^to 10^th ^grade, respectively) and two-week test-retest reliability of 0.86 (ICC) for the ILC LQ0-100 score [37]. The parent version has also shown satisfactory internal consistency and test-retest reliability [37].

#### The Kinder Lebensqualität Fragebogen (KINDL)

The KINDL [14,15] is a QoL measure developed for the assessment of children and adolescents both in the general population and clinical samples. Here, the 8–12 and 13–16 year age forms were used as well as a proxy version completed by the parents. The forms consist of 24 items equally distributed into the following six subscales: Physical well-being, emotional well-being, self-esteem, family, friends, and school. Each item addresses the child's experiences over the past week and is rated on a 5-point scale (1 = never, 5 = always) with item 1–3, 6–8, 15–16, 20 and 23–24 scores reversed. Mean item scores are calculated for all subscales and the total scale, which are transformed to a 0–100 scale, 0 indicates very low and 100 very high QoL. Correlations with comparable QoL scales [16] have shown acceptable convergent validity as well as satisfactory discriminant validity [15]. In regard to sensitivity, the KINDL showed significant changes after a six-week inpatient rehabilitation program for chronically ill children (effect sizes from d = .02 to .69, and .24 for the total QoL scale and the whole sample) [41]. In the original German version, Cronbach's alpha was approximately .70 for most subscales, while the overall scale had an alpha value over .80. In the Norwegian version [37], generally satisfactory alpha values were found (from .64 to .81 for the subscales, and .83 to .89 for the total scale and children in the 4^th ^to 10^th ^grades). However, low alpha values were obtained for the School, Friends, and Emotional well-being subscales and 4^th ^graders. Except for the physical well-being scale (ICC = .43), two-week test-retest coefficients were good to excellent (ICC from .70 to 87). The Parent version showed satisfactory alpha values and test-retest reliability [37].

The ILC and the KINDL measures were developed for different research and clinical purposes, they differ in items, content and length. To gain a comprehensive picture of various aspects of short-term changes in QoL among school children in our investigation, we used both instruments.

Socio-demographic information on age and sex was obtained from the students.

### Ethics

Before students could participate in the study, their parents had to give their written consent. The Norwegian Ethical Committee of Medical Research and the Norwegian Data Inspectorate approved of the research protocol.

### Statistical analysis

Missing values were substituted by using expectation maximization (EM) procedures. Group means were compared by independent t-test or ANOVA. Differences between baseline (T1) and retest raw scores (T2) were calculated (by subtracting T1 from T2 scores) and ranged from -100 to +100. These scores were used as dependent variables in ANCOVA with T1 scores as covariates. Effect sizes for between-group differences were calculated by means of eta squared (ES) as recommended by Cohen [42]. An alpha level of p < 0.05 indicated statistical significance, except for overall main and interaction effects in multiple ANCOVA in which an alpha of < 0.01 was set due to multiple comparisons. All ANCOVA involving more than two groups were conducted using "repeated contrasts", i.e., one group was compared to its preceding group and the next group, with a hypothesis of linearity of age-related means. Possible cluster effects have previously been examined in the baseline study [37] by means of Mixed Linear Models. The results showed that only 3.6% of the total variance of the ILC LQ 100 scores, and 6.5% of the total KINDL Total QoL scores could be explained by differences *between *the cohorts in the study.

## Results

**Figure 2:**
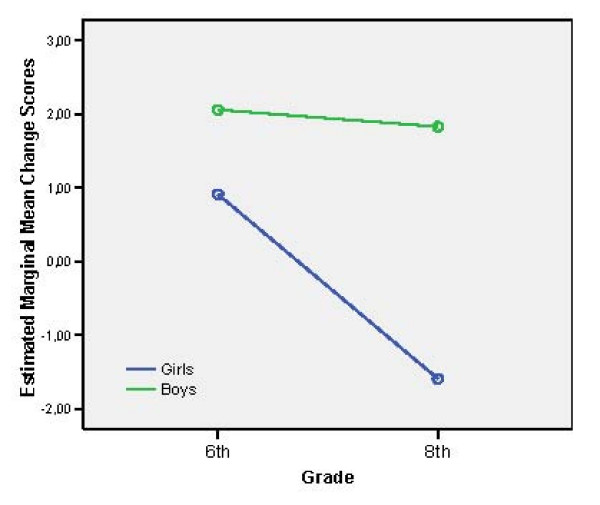
**Grade and sex interaction effect on the ILC across the 6-month follow-up**.

Descriptive information on the various KINDL subscales and on the ILC are presented in Tables 2, 3 and 4. Mean change scores (i.e. means of differences in raw scores between baseline and follow-up) and results of ANCOVA are shown in Tables 5, 6 and in Figure 2. It should be noted that corrected mean changes in baseline-follow-up differences were obtained in ANCOVA using baseline scores as covariates.

**Table 2:** Mean raw scores on KINDL subscales: Student report by grade

	**Mean**		**Standard deviation**	
		
**Grade**	**T1**	**T2**	**T1**	**T2**
Family				
10^th^	71.5	72.1	20.3	19.7
8^th^	76.2	74.8	17.7	19.1
6^th^	79.4	81.7	15.1	15.2
4^th^	81.7	82.7	16.1	14.1
^1^Total	77.2	77.8	17.9	17.7

Friends				
10^th^	73.2	74.4	16.6	16.2
8^th^	74.9	75.9	15.7	16.4
6^th^	77.4	78.9	16.6	16.3
^2^Total	75.1	76.4	16.4	16.4

School				
10^th^	58.6	60.2	19.3	18.6
8^th^	65.6	65.2	17.6	16.9
6^th^	70.1	70.3	16.5	16.7
^2^Total	64.4	65.0	18.6	18.0

Emotional well-being				
10^th^	74.0	74.1	16.0	16.3
8^th^	77.4	77.5	14.1	15.5
6^th^	77.1	80.5	14.3	13.1
^2^Total	76.0	77.2	15.0	15.3

Self-esteem				
10^th^	53.7	54.3	19.2	20.4
8^th^	56.2	55.3	19.7	21.3
6^th^	57.4	57.8	18.8	19.0
4^th^	56.0	55.8	20.5	21.1
^1^Total	55.7	55.8	19.6	20.5

Sample size for 10^th^, 8^th^, 6^th^, 4^th ^grade at T1: 494, 374, 434, 488.

Sample size for 10^th^, 8^th^, 6^th^, 4^th ^grade at T2: 493, 377, 437, 490.

T1 = at baseline; T2 = at 6-month follow-up

^1^Total N : T1 = 1790; T2 = 1797.

^2^Total N : T1 = 1302; T2 = 1307.

**Table 3:** Mean raw scores on KINDL subscales: Parent proxy report by grade

	**Mean**		**Standard deviation**	
		
**Grade**	**T1**	**T2**	**T1**	**T2**
Family				
10^th^	74.7	76.8	13.4	12.7
8^th^	75.7	76.6	12.7	12.1
6^th^	75.3	76.5	12.6	13.1
4^th^	75.3	77.1	12.0	11.8
Total	75.3	76.8	12.4	11.6

Friends				
10^th^	77.4	78.7	12.8	12.0
8^th^	78.8	78.8	12.6	12.0
6^th^	77.5	78.7	13.7	11.9
4^th^	80.1	81.3	11.8	10.8
Total	78.6	79.6	12.7	11.6

School				
10^th^	72.1	72.5	14.7	15.6
8^th^	73.8	75.1	14.2	12.5
6^th^	75.9	77.5	18.9	12.8
4^th^	81.6	81.1	11.2	12.1
Total	76.6	77.2	15.3	13.5

Emotional well-being				
10^th^	79.3	80.4	13.4	13.1
8^th^	79.6	81.2	13.5	12.7
6^th^	78.4	80.3	13.8	13.4
4^th^	80.6	81.7	11.5	10.9
Total	79.6	80.9	12.9	12.4

Self-esteem				
10^th^	65.0	66.6	14.8	13.5
8^th^	66.4	66.9	14.0	12.7
6^th^	66.0	65.8	14.0	14.0
4^th^	70.1	70.1	12.6	12.2
Total	67.2	67.6	13.8	13.2

Sample sizes for 10^th^, 8^th^, 6^th^, 4^th ^grade and total at T1: 266, 268, 349, 436 and 1319.

Sample sizes for 10^th^, 8^th^, 6^th^, 4^th ^grade and total at T2: 267, 271, 352, 436 and 1326.

T1 = at baseline; T2 = at 6-month follow-up

**Table 4:** Mean raw scores on the ILC: Student and parent proxy report by sex and grade

	**Girls**						**Boys**					
		
	**Mean**		**Standard deviation**		**Sample size**		**Mean**		**Standard deviation**		**Sample size**	
						
Grade	T1	T2	T1	T2	T1	T2	T1	T2	T1	T2	T1	T2
Student report												
10^th^	74.5	76.3	15.2	15.8	260	260	80.8	81.1	13.4	14.0	240	240
8^th^	80.1	78.9	12.9	15.7	187	187	82.9	84.2	12.7	13.3	196	195
6^th^	81.6	82.4	14.7	13.6	231	231	80.7	82.9	15.5	14.6	212	215
4^th^	82.2	82.9	11.1	11.5	231	235	84.2	82.3	11.6	11.4	254	255
Total	79.4	80.1	14.0	14.5	909	913	82.2	82.5	13.4	13.3	902	905

Parent proxy report												
10^th^	86.7	87.9	13.6	12.7	151	151	86.6	87.1	12.5	13.5	121	121
8^th^	88.1	87.5	9.6	11.3	141	142	84.4	86.3	12.6	11.6	130	129
6^th^	87.3	88.6	11.0	10.8	180	180	83.0	84.6	14.2	13.0	173	172
4^th^	88.9	90.0	9.8	9.0	214	213	87.0	88.4	10.9	10.5	223	223
Total	87.8	88.6	11.0	10.9	686	686	85.4	86.7	12.6	12.1	647	645

T1 = at baseline

T2 = at 6-month follow-up

**Table 5:** Mean change and estimated mean change on the KINDL: Student report by grade

	**Mean change**^a^	**SD**	**Est. Mean change**^b^	**SEM**	**Effect size (%)**
	
Grade					
Family					
10^th^	0.4	18.6	-2.3	0.7	3.3
8^th^	-1.7	16.2	-2.2***	0.8	
6^th^	2.1	14.9	3.1***	0.7	
4^th^	1.0	16.7.	3.2	0.7	
Total^c^	0.5	16.8	-	-	

Friends					
10^th^	1.1	14.8	0.2	0.6	
8^th^	1.0	15.4	0.8	0.7	
6^th^	1.4	15.7	2.4	0.7	
Total^d^	1.2	15.2	-	-	

School					
10^th^	1.4	16.9	-1.0	0.7	1.0
8^th^	-0.5	15.7	-0.1*	0.7	
6^th^	-0.3	15.1	2.2*	0.7	
Total^d^	0.3	16.0	-	-	

Emotional well-being					
10^th^	0.1	15.3	-0.9*	0.6	2.2
8^th^	0.3	14.8	0.9*	0.7	
6^th^	3.2	15.0	3.8*	0.6	
Total^d^	1.2	15.1	-	-	

Self-esteem					
10^th^	0.5	15.6	-0.3	0.8	
8^th^	-0.9	18.6	-0.7	0.9	
6^th^	0.3	16.9	1.0	0.8	
4^th^	-0.3	23.4	-0.3	0.8	
Total^c^	-0.1	18.9	-	-	

^a^Differences in means based on raw scores between baseline and follow-up (T2 minus T1).

^b^Estimated marginal mean change scores by ANCOVA, using baseline-scores as covariates.

^c^Total N = 1770; ^d^total N= 1282.

Sample size for 10^th^, 8^th^, 6^th ^and 4^th ^grades: 488, 370, 424 and 488.

SD = Standard deviation; SEM = Standard error of the mean

*p < 0.05; **p < 0.01; ***p < 0.001

**Table 6:** Mean change and estimated mean change on the KINDL: Parent proxy report by grade

	**Mean change **^a^	**SD**	**Est. Mean change**^b^	**SEM**
	
Grade				
Family				
10^th^	1.8	11.3	1.5	0.6
8^th^	1.0	12.1	1.2	0.6
6^th^	1.3	12.2	1.3	0.5
4^th^	1.9	11.1	1.8	0.5
Total	1.5	11.6	-	-

Friends				
10^th^	1.2	11.5	0.7	0.6
8^th^	-0.1	11.1	0.1	0.6
6^th^	1.3	11.7	0.8	0.5
4^th^	1.3	10.5	2.0	0.5
Total	1.0	11.2	-	-

School				
10^th^	0.3	11.5	-2.1*^c^	0.7
8^th^	1.3	11.5	-0.1*	0.7
6^th^	1.6	17.9	1.2	0.6
4^th^	-0.5	11.1	2.1	0.5
Total	0.6	13.4	-	-

Emotional well-being				
10^th^	1.2	12.2	0.7	0.7
8^th^	1.4	12.1	1.5	0.7
6^th^	2.0	13.7	1.4	0.3
4^th^	1.3	12.8	1.7	0.5
Total	1.4	12.8	-	-

Self-esteem				
10^th^	1.4	12.8	0.3	0.7
8^th^	0.5	13.7	0.2	0.7
6^th^	-0.1	14.1	-0.8	0.6
4^th^	0.1	11.3	1.4	0.5
Total	0.4	12.9	-	-

^a^Differences in mean change based on raw scores between baseline and follow-up (T2 minus T1).

^b^Estimated marginal mean change scores by ANCOVA, using baseline-scores as covariates.

^c^Effect size = 2%

SD = Standard deviation; SEM = Standard error of the mean

Sample size for 10^th^, 8^th^, 6^th^, 4^th ^grades and total: 262, 266, 348, 434 and 1310.

*p < 0.05

### Student report

#### Family-related QoL

Across the six-month follow-up a significant main effect for grade, [F (3, 1761) = 19.86, p < 0.001] was found (ES = 3.3%). Subsequent posthoc comparisons showed that children in the 8^th ^grade reported a significantly (p < 0.001) greater decrease in family-related QoL than did those in the 6^th ^grade over the six-month follow-up period (see Table 5).

#### Friends

No significant effect for sex, grade or grade by sex interaction was observed (see Table 5).

#### School

Across the six-month follow-up period, a significant main effect for grade, [F (2, 1275) = 5.57, p < 0.01] (ES = 1%) was found. Subsequent posthoc comparisons showed that children's reported QoL in respect to school in 8^th ^grade decreased significantly (p < 0.05) more during the follow-up period as compared to those in 6^th ^grade (see Table 5).

#### Emotional well-being and self-esteem

Across the six-month follow-up period, a significant main effect for grade [F (2, 1275) = 14.67, p < 0.001] (ES = 2.2%) was observed for emotional well-being. In subsequent posthoc comparisons a significant linear decrease was found, in that the emotional well-being of children in the 10^th^grade decreased (p < 0.05) more than those in the 8^th ^grade, while the emotional well-being of the latter decreased (p < 0.01) more than that of children in the 6^th ^grade (see Table 5).

While a non-significant main effect for grade was found for self-esteem, a significant main effect was observed for sex, [F (1, 1761) = 10.08, p < 0.01] (ES = 0.6%) in that girls' self-esteem decreased more than boys' over the six-month follow-up (the estimated mean change score for girls was -1.4 (SEM = 0.6) vs. boys 1.2 (SEM = 0.6)).

#### Total QoL

On the total QoL KINDL score a significant main effect for grade, [F (3, 1761) = 10.59, p < 0.001] (ES = 2%) was found. The overall QoL of children in the 8^th ^grade decreased significantly (p < 0.001) more than for those in the 6^th ^grade. Mean raw scores for girls reports on the ILC LQ0-100 score were higher for 4^th ^and 6^th ^graders than for 8^th ^and 10^th ^graders, while differences for boys were neglectable (see Table 4). A significant sex by grade interaction effect was found, [F (3, 1799) = 4.16, p < 0.01] (ES = 0.7%). Further analysis showed that overall QoL levels for girls in the 8^th ^grade decreased significantly [F (1, 819) = 8.25, p < 0.01] more than for those in the 6^th ^grade over the six-month follow-up period as compared to boys, whose QoL scores remained stable across 6^th ^and 8^th ^grade (see Figure 2).

### Parent report

One significant main effect was observed on the KINDL school scale for grade, [F (3, 1301) = 8.15, p < 0.001] (ES = 2%). Subsequent post hoc tests showed that children's attitude to school in the10^th ^grade as perceived by their parents, decreased significantly (p < 0.05) more during the follow-up period as compared to those in 8^th ^grade (see Table 6).

### Ceiling effects

The proportions of students who reported maximum scores at baseline assessment on the KINDL subscales were the following: Emotional well-being 3.8%, Friends 6.1%, School 2.2%, Family 11.8% and Self-esteem 1.8%. The corresponding values for parent proxy report were: Emotional well-being 3.3%, Friends 5.6%, School 4.9%, Family 5% and Self-esteem 1.7%. For the ILC LQ0-100, the respective values were 7% for student and 13.7% for parent proxy report.

## Discussion

The present study of short-term changes in child- and parent reports of child QoL in a representative school-based sample of Norwegian students, aged 8–16 years, showed statistically significant differences related to age and sex in various domains. Students in the 8^th ^grade reported a decrease in QoL over the six-month follow-up period as compared to those in the 6^th ^grade with regard to the QoL Family, School domains and total QoL. For emotional well-being, a significant linear decrease in QoL levels across grades 6^th ^to 10^th ^was observed over the follow-up period. However, student ratings on the Friends and Self-esteem domains did not change significantly by age. Girls reported a higher decrease in their QoL across all age-groups over the follow-up period than did boys in respect of Self-esteem, as well as an age-related decrease in total QoL between 6^th ^and 8^th ^grade. Parents reported significant changes of child QoL across the six months only for the School-domain. Overall, all significant changes reported by students and parents showed small effect sizes.

### Age-related effects

#### Developmental trends in QoL related to family and friends

The results supported our first hypothesis. The decrease over six months as reported by the students in family-related QoL between 6^th ^and 8^th ^grade is likely to reflect a desire for increased autonomy in early adolescence and puberty. Our results are in accordance with a two-year follow-up study [5] showing that adolescent reports of affection towards parents declined, for fathers from the 6^th ^to 8^th ^grades and for mothers from the 8^th ^to 10^th ^grades. The adolescents also reported a decrease in reports of helpfulness towards their parents. Larson et al. [3], observed signs of transformation in adolescents' changing emotional experience with their families. The emotional states among early adolescents became less positive, especially during talk with their families, when they experienced family members as less friendly. The authors concluded that early adolescence is often the most strained period in adolescent-parent relationships [3,43]. While it is likely that our results also reflect such transformations in adolescents-parent relationships, it is notable that the parents did not report similar child QoL changes in this domain. The students' report could have been influenced by their emotions and need for autonomy rather than reflect real changes in family conflict. A similar conclusion was drawn by Eberly and Montemajor [5] who found that parents did not report the same developmental changes in adolescents' affection or helpfulness obtained on adolescent report. Thus, it is likely that parents may have difficulties in detecting minor changes in their child's feelings over short-term, or they perceive the emotional fluctuations in their children as a normal phenomenon.

As expected, students perceived their relationships with friends as stable across age over the six-month follow-up period. In their review, Hartup and Stevens [9] concluded that good outcomes in respect to mental health are most likely when a child is well socialized and has friends, and when relationships with these individuals are supportive and intimate. Thus, the high degree of stability related to the QoL Friends domain in our school sample may reflect normal development among adolescents. Parent proxy reports further supported stability in student perception of relationships with friends.

#### Discontinuity in school-bonding and QoL

Our hypothesis that reports of older students on school QoL would decrease during the follow-up period, as compared to younger ones was supported. The overwhelming majority of the 8^th ^graders had recently moved to junior high school, representing a discontinuity in their school situation. Wigfield et al. [11] found that self-perceived ability in mathematics, English, sports and social activities declined after transition from elementary school to junior high school (6^th ^to 7^th ^grade in USA) possibly due to changes in school and classroom environments. They also observed a temporary decline of self-esteem among students associated with the transition. Norwegian children receive marks for the first time in the 8^th ^grade, a potential school stressor that may also have impact on school-related QoL. The discontinuities in student school bonding may explain some of the observed decrease in school-related QoL between the 6^th ^and 8^th ^grades. This domain was the only one in which parents reported significant changes across the six-month follow-up period. This finding supported our hypothesis that parent by proxy ratings will show fewer significant age and sex-related changes in child QoL over the six-month follow-up period than student reports. Parents also reported a decrease of QoL between the 8^th ^and 10^th ^grade, i.e. somewhat later than did the students, an unclear finding. The reason why the only parent-reported change was restricted to the School domain, might be the existence of objective information such as marks and teacher reports providing the parents with some external indication about student's school-related QoL. Regardless of the exact time period of change, both students and parents in our school sample reported a decrease in school-related Qol with increasing student age.

#### Developmental trends in emotional well-being and Self-esteem

The hypothesis that older students would report a decrease in emotional well-being as compared to younger ones over the follow-up period was supported. Pubertal changes combined with challenges for the maturing adolescent in social contexts, e.g. in the family, school, is likely to affect his/her emotional well-being from early to mid-adolescence [44]. The observed linear decrease in student reports of emotional well-being across the 6^th^, 8^th ^and 10^th ^grades represent a small effect and reflects an age-related, temporary instability of emotional well-being among the students as part of their normal psychological development. Emotional well-being was shown to be the only domain, in which 10^th ^graders also reported a decrease in QoL. It might be that other pubertal factors not investigated in the present study, were responsible for the decrease in emotional well-being among10^th ^graders, i.e. love relationships.

By contrast, parents did not detect any significant age-related changes in regard to their child's emotional well-being. From comprehensive cross-informant studies on child emotional and behavioural problems [45], it is well known that child-parent correlations in reports of internalizing problems are lower than overt behaviour problems.

As expected, differences between the four grades in students' reports of self-esteem across the six-month follow-up period, were small and nonsignificant. In their extensive meta-analyses of 50 published studies (N = 29,839) and four large national studies (N = 74,381), Trzesniewki et al. found evidence for a robust developmental trend. The stability of self-esteem was low during childhood (up to the age of 9 years), increased throughout adolescence into young adulthood and declined during midlife and old age [17]. Overall, the authors concluded that self-esteem is a stable trait across adolescence.

### Sex-related effects

#### QoL and sex-related developmental changes

While the ILC evidenced a decrease of total QoL scores between the 6^th ^and 8^th ^grade across the six-month follow-up period, it was only shown for girls. Although such sex by age interaction effect was not observed on the KINDL total QoL scale, girls scored significantly lower across all grades on the KINDL self-esteem subscale. Our results support the hypothesis that girls will report a lower total QoL than boys. In a 10-year longitudinal study, Biro et al. [19] found that only Caucasian girls, as compared to Afro-American girls showed a decline in self-esteem during adolescence. The findings are also in line with other cross-sectional studies showing that girls report a lower total QoL than boys [20,21]. Even if the student reported sex by age differences in our study were small, it is notable that they were obtained after a 6-month follow-up period. However, parents did not report sex-related QoL changes among students on any subscale or for total QoL scores.

### Implication of the findings for clinical research and practice

(1) The present study illustrates the importance of obtaining both child and parent proxy reports when assessing QoL changes, in epidemiological surveys as well as in clinical populations. The informants provide different perspectives and parent proxy report can not substitute for child or adolescent subjective evaluation. (2) Only a QoL instrument should be used that includes a generic part with norms available in the general child population in regard to age and sex. (3) When using QoL as an outcome measure in clinical practice or research, the clinician should expect a natural decrease across 6 months in QoL related to family and emotional well-being domains in the 12 to 14 (15) year age group. (4) With regard to the child's school-related QoL, the clinician should assess recent or future stressors in school that might implicate a discontinuity in school-bonding. (5) Clinicians should also be aware of a greater decrease in QoL among girls than boys in puberty.

### Strengths and Limitations of the study

The present follow-up sample was found to be representative for the population with regard to urban-to-rural residency ratio, sex ratio, and mean age. Because the two-week test-retest reliability of the reported KINDL scales and the ILC was overall good to excellent [37], we can be confident in that our results reflect real QoL changes across the 6-month period in respect to student age and sex.

Because four KINDL subscales in a former study showed low reliability (internal consistency or two-week test-retest reliability) for the youngest children in the 4^th ^grade [37], they were not included in all analyses here, limited to the 6^th ^to 10^th ^grades. Further, parents who did not participate at the follow-up reported a slightly, but significantly lower QoL in their children at baseline as compared to participants. Thus, our follow-up figures for parent reports of child QoL may therefore be slightly overestimated. Overall, we found small to moderate ceiling effects. The highest ceiling effects were found for the student report on the KINDL Family-subscale and for the parent proxy report on the ILC LQ0-100 scale. Thus, the observed differences in QoL for 8^th ^graders compared to 6^th ^graders over the six-month follow-up on the KINDL family scale and the corresponding effect size, might therefore be slightly underestimated. Similarly, student and parent reports of stability on the Friends subscale, and parent report on the ILC LQ0-100 scale could be slightly biased due to moderate ceiling effects.

## Conclusion

The child-reported changes in various QoL domains represented small effects and could be interpreted as reflecting normal psychological developmental during puberty, involving cognitive and emotional changes and contextual transitions in parent-child relationships, friends and school domains. However, it is important to be aware of short-term changes of QoL among children and adolescents in the general population, in particular in puberty. Such aspects are important considerations when assessing changes in QoL in clinical populations.

## Abbreviations

ANOVA: Analysis of variance; ANCOVA: Analysis of covariance; EM: Expectation maximization; ES: Effect size; ICC: Intraclass correlation coefficient; ILC: Inventory of Life Quality for Children and Adolescents; KINDL: Kinder Lebensqualität Fragebogen (In German. Questionnaire for Measuring health-related Quality of life in children and adolescents); LQ0-100: Life quality score (range 0–100); SEM: Standard error of the mean; T1: time 1; T2: time 2; QoL: Quality of Life.

## Competing interests

The authors declare that they have no competing interests.

## Authors' contributions

TJ contributed to the study design, data collection, statistical analysis, interpretation of data and to the drafting of the paper. BL contributed to the study design, statistical analysis, interpretation of data and the revision of the manuscript. LW contributed to the study design, statistical analysis, interpretation of data and revision of the manuscript. All authors read and approved the final manuscript.
